# Attitude towards and perception of individual safety after SARS-CoV-2 vaccination among German cancer patients

**DOI:** 10.1007/s00432-022-04099-7

**Published:** 2022-06-22

**Authors:** Oliver Overheu, Simon Lendowski, Daniel R. Quast, Corinna S. Marheinecke, Eleni Kourti, Celine Lugnier, Ioana Andreica, Uta Kiltz, Stephanie Pfaender, Anke Reinacher-Schick

**Affiliations:** 1 Department of Hematology and Oncology with Palliative Care, St. Josef Hospital, Ruhr University Bochum, Gudrunstr 56, 44791 Bochum, Germany; 2 Department of Internal Medicine, St. Josef Hospital, Ruhr University Bochum, Bochum, Germany; 3 Department of Molecular and Medical Virology, Ruhr University Bochum, Bochum, Germany; 4 Rheumazentrum Ruhrgebiet, Ruhr University Bochum, Herne, Germany

**Keywords:** SARS-CoV-2, COVID-19, Vaccination, Hesitancy, Cancer, Tolerability

## Abstract

**Purpose:**

Refusal to receive SARS-CoV-2 vaccination poses a threat to fighting the COVID-19 pandemic. Little is known about German cancer patients’ attitude towards and experience with SARS-CoV-2 vaccination.

**Methods:**

Patients were enrolled between 04–11/2021. They completed a baseline questionnaire (BLQ) containing multiple choice questions and Likert items ranging from 1 (“totally disagree”) to 11 (“totally agree”) regarding their attitude towards vaccination and COVID-19. A follow-up questionnaire (FUQ) was completed after vaccination.

**Results:**

218 patients (43% female) completed BLQ (110 FUQ; 48% female). Most patients agreed to “definitely get vaccinated” (82%) and disagreed with “SARS-CoV-2 vaccination is dispensable due to COVID-19 being no serious threat” (82%; more dissent among men, *p* = 0.05). Self-assessment as a member of a risk group (*p* = 0.03) and fear of COVID-19 (*p* = 0.002) were more common among women. Fear of side effects was more common among women (*p* = 0.002) and patients with solid or GI tumors (*p* = 0.03; *p* < 0.0001). At FUQ, almost all (91%) reported their vaccination to be well tolerated, especially men (*p* = 0.001). High tolerability correlated with confidence in the vaccine being safe (*r* = 0.305, *p* = 0.003). Most patients would agree to get it yearly (78%). After vaccination, patients felt safe meeting friends/family (91%) or shopping (62%). Vacation (32%) or work (22%) were among others considered less safe (less frequent among men, *p* < 0.05).

**Conclusion:**

Acceptance of SARS-CoV-2 vaccination is high and it is well tolerated in this sensitive cohort. However, concerns about vaccine safety remain. Those and gender differences need to be addressed. Our results help identify patients that benefit from pre-vaccination consultation.

## Introduction

The severe acute respiratory syndrome virus type 2 (SARS-CoV-2) and its corresponding coronavirus disease 2019 (COVID-19) pandemic remain a worldwide health issue (Ioannidis 2020; Wu et al. 2020). Especially immunocompromised patients, e.g. cancer patients, are at an increased risk of a severe course of COVID-19 (Dai et al. 2020; Lee et al. 2020; Williamson et al. 2020; Rüthrich et al. 2021). As serological prevalence of SARS-CoV-2 antibodies remains low among cancer patients (Overheu et al. 2022), newly established SARS-CoV-2 vaccines offer protection by effectively reducing the risk of a severe course (Polack et al. 2020; Baden et al. 2021; Voysey et al. 2021; Fendler et al. 2022).

Therefore, national vaccination programmes were implemented worldwide in 2021 to achieve herd immunity. However, refusal to receive a SARS-CoV-2 vaccination poses a serious threat to those global efforts in fighting the COVID-19 pandemic. Vaccine hesitancy has already been labeled as one of ten threats to global health by the World Health Organization (WHO) in 2019 (WHO 2019). Reasons for refusal of SARS-CoV-2 vaccination include concerns about its safety as well as both mis- and disinformation (Jaiswal et al. 2020; Lindholt et al. 2021; Thunström et al. 2021; Pertwee et al. 2022). Additionally, rates of and reasons for acceptance or hesitancy differ between countries or social groups and are therefore difficult to assess (Heyerdahl et al. 2022; Shakeel et al. 2022). While acceptance of SARS-CoV-2 vaccination is generally high among cancer patients (Barrière et al. 2021; Brodziak et al. 2021), it is overall rather moderate in Germany (Neumann-Böhme et al. 2020; Bendau et al. 2021; Holzmann-Littig et al. 2021; Lindholt et al. 2021; Umakanthan and Lawrence, 2022). However, little is known about the attitude of German cancer patients towards and their experience with the SARS-CoV-2 vaccination (Heyne et al. 2022).

## Methods

Cancer patients at our academic cancer center were prospectively enrolled on this study between April and November 2021. After obtaining informed consent they were asked to complete a self-created baseline questionnaire containing multiple-choice questions and ten eleven-level Likert items. Those range from 1 (“totally disagree”) to 11 (“totally agree”) and regard the patients’ attitude towards vaccinations and especially anti-SARS-CoV-2 vaccination and COVID-19 as well as their medical history. Questionnaires were complemented by data from patients’ medical files. A follow-up questionnaire, including questions about the tolerability and safety of the vaccine as well as attitude changes towards COVID-19 and its vaccination, was completed after SARS-CoV-2 vaccination or already at baseline if patients have previously been vaccinated.

The study was approved by the Ethics Committee of the Medical Faculty, Ruhr University Bochum (reference number 20–6953-bio and 21–7351) and conducted in accordance with the Declaration of Helsinki. Descriptive data are presented as *n* (%) or median. All own percentual results are rounded to the nearest full number. Data were analyzed using Welch’s and student’s *t*-test, respectively, chi-squared test or Spearman’s correlation coefficient test using SPSS (v. 26). Results were considered significant at *α* = 0.05.

## Results

### Patient characteristics

Overall, 218 patients were enrolled on this study and completed the baseline questionnaire. 110 patients completed the follow-up questionnaire. The baseline characteristics are presented in Table 1. Mean age was 64 (24–87) years, 43% of patients were female (48% at follow-up). The majority of patients suffered from solid tumors (82%), mainly gastrointestinal cancer (56%). Most patients were on active cancer therapy (93%), mainly chemotherapy (80%). Nine patients (4%) previously had COVID-19, and none of them had experienced a severe course. Twenty-nine patients personally knew someone who had died from COVID-19. At the time of the study most patients (78%) had already received at least one COVID-19 vaccine, mainly BNT162b2 (87%) or ChAdOx1-S (10%), 131 patients (60%) had already been vaccinated twice.

**Table 1:** Patients' characteristics

Median age	64 (24–87)
Sex	
Female	94 (43%)
Male	124 (57%)
Cancer type	
Pancreas	86 (40%)
Colorectal	22 (10%)
Gastric/Esophageal	9 (4%)
Hepatobiliary	5 (2%)
Lung	30 (14%)
Lymphoma	16 (7%)
Leukemia	11 (5%)
Myeloma	12 (6%)
Gynecological	7 (3%)
Head and Neck	9 (4%)
Other	8 (4%)
Patients on active cancer treatment	203 (93%)
Chemotherapy	174 (80%)
Radiation	38 (18%)
Immunotherapy	30 (13%)
Targeted therapy	21 (10%)
B cell depleting therapy	6 (3%)
History of COVID-19	9 (4%)
Already vaccinated against SARS-CoV-2 at baseline	
Once	170 (78%)
Twice	131 (60%)

Data are presented as *n* (%) or median (range)

### Baseline questionnaire

**Fig. 1:**
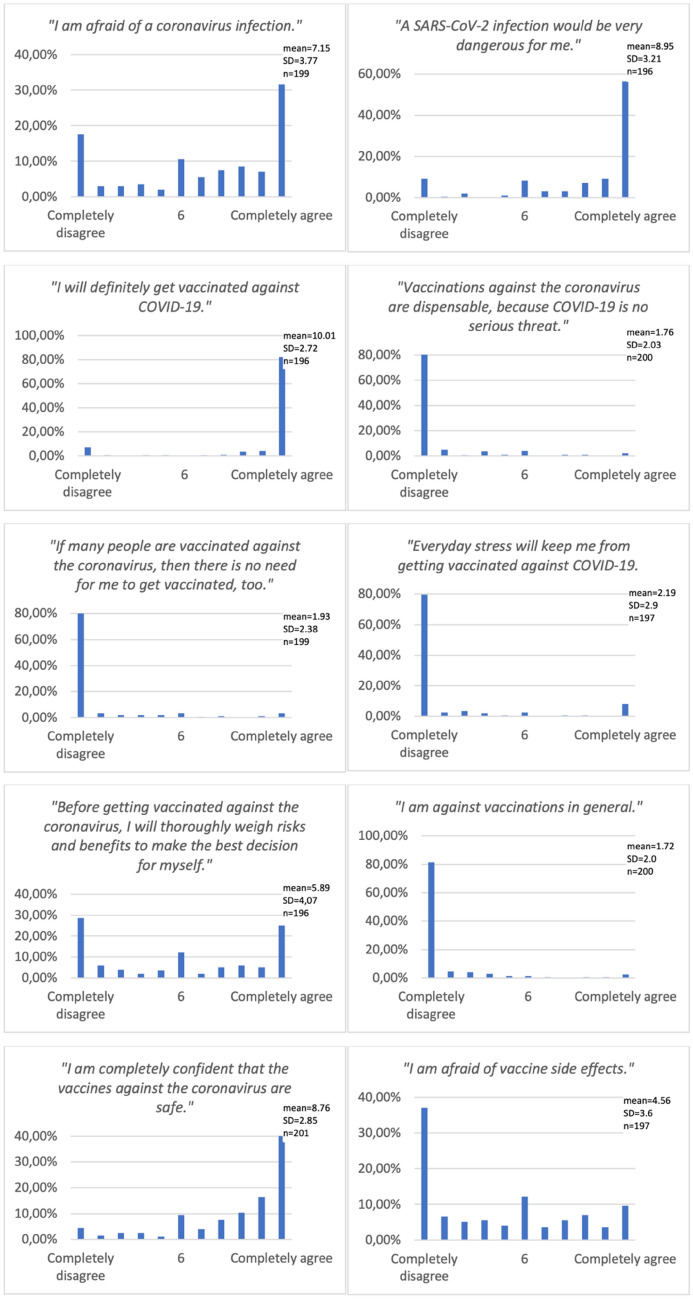
Patients' overall responses on Likert items ranging from 1 (“Completely disagree”) to 11 (“Completely agree”)

**Fig. 2:**
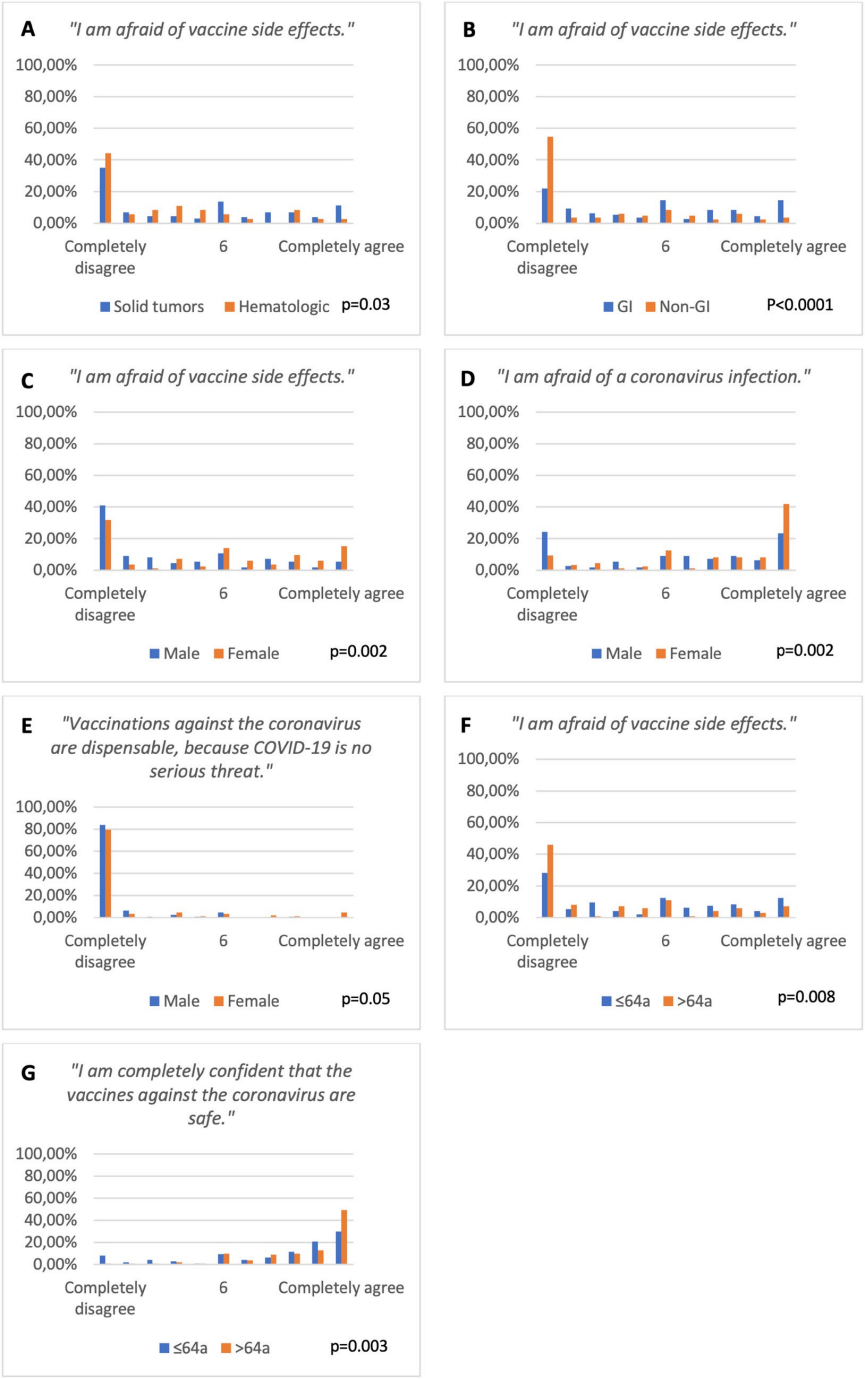
Responses on Likert items with a focus on items with significant differences in mean response between groups, either sorted by tumor type (**A**, **B**), gender (**C**-**E**) or age (**F**, **G**)

While only 16 patients (7%) declared not to favor any specific vaccine, most favored BNT162b2 (55%) or mRNA-1273 (11%). One percent refused vaccination. All responses on the Likert scale are presented in Fig. 1, those Likert items with significant differences between groups are displayed in Fig. 2. Most patients (82%) completely agreed to “definitely get vaccinated” as well as completely disagreed with “vaccination being dispensable due to COVID-19 being no serious threat” (82%; statistically significant more dissent among men, *p* = 0.05) and “being against vaccinations in general” (82%). Every third patient completely agreed to “being afraid of COVID-19” (31%), every second thinks “SARS-CoV-2 infection would be very dangerous” (56%). Self-assessment as member of a risk group (61% vs. 46%, *p* = 0.03) and fear of COVID-19 (*p* = 0.002) were statistically significant more common among women.

However, only 41% of patients expressed “complete confidence in the vaccine being safe” as well as 37% “not being afraid of side effects”. Fear of vaccine side effects was more common among women (*p* = 0.002), patients with solid tumors (*p* = 0.03), with GI tumors (*p* < 0.0001) and those below mean age (≤ 64 years, *p* = 0.008). The latter accordingly expressed significantly less “confidence in the vaccine being safe” (*p* = 0.003).

While 54% of patients reported a history of influenza vaccination within the last five years, only 28% received a pneumococcal vaccine within the same period. Interestingly, those without any of those vaccinations within the last five years expressed less fear of SARS-CoV-2 vaccine side effects, although not significantly (*p* = 0.087).

### Follow-up questionnaire

At the follow-up questionnaire, most patients (91%) reported their SARS-CoV-2 vaccination to be well tolerated, 44% reported no side effects at all, especially men (*p* = 0.001) and patients above age average of 64 years (*p* = 0.002). Most common side effect was local pain at injection site (35%), which was more frequent among women (*p* = 0.002), younger patients (*p* = 0.024) and patients with solid tumors (*p* = 0.04). Other common side effects included fatigue (18%) and myalgia (8%). No thromboembolic or other serious adverse events that would have required hospital admission occurred. Only three patients had their therapy postponed due to the side effects of SARS-CoV-2 vaccination. Just one patient needed to seek medical assistance due to side effects. Patients were mainly vaccinated by their general practitioner (44%) or at local vaccination centers (45%). Almost all patients felt retrospectively sufficiently informed about their vaccination and possible side effects (94%), would have it again (88%) and agree to get it yearly, if recommended (78%). High tolerability was significantly correlated with patients’ confidence in the vaccine being safe (*r* = 0.305, *p* = 0.003).

**Fig. 3:**
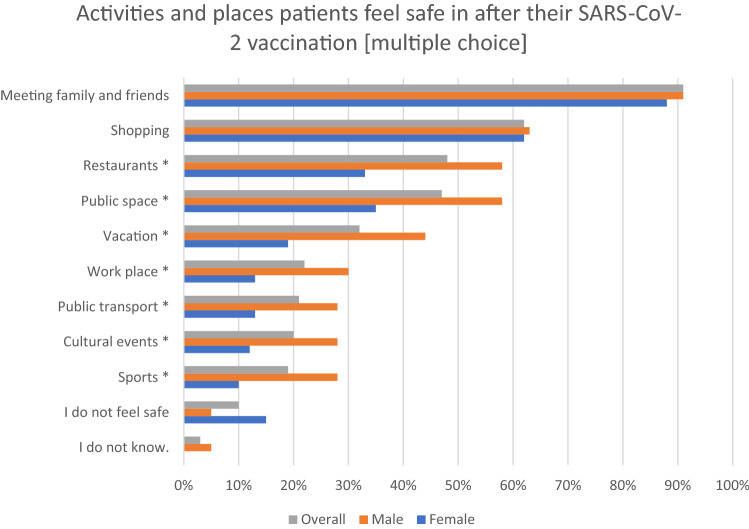
Activities and places patients feel safe in after their SARS-CoV-2 vaccination [multiple choice]. An asterisk * indicates a significant difference between male and female patients, *p* < 0.05

After SARS-CoV-2 vaccination, patients felt safe meeting friends or family (91%) or shopping (62%; see Fig. 3). Restaurants (48%), public space (47%), vacation (32%), work (22%), public transport (21%), cultural events (20%) or sports (19%) were considered less safe (less frequent among men, *p* < 0.05). Only 10% of patients declared not to feel safe despite SARS-CoV-2 vaccination. Most patients (70%) did not feel that the COVID-19 pandemic negatively influenced their cancer treatment and regarded the hospitals protective measures (e.g., mandatory protective masks, pre-admission SARS-CoV-2 testing) as adequate (91%).

## Discussion

Our results demonstrate that willingness to get a SARS-CoV-2 vaccination is high among German cancer patients and SARS-CoV-2 vaccination is well tolerated in this sensitive cohort. This is in line with previously published national (Heyne et al. 2022) and international (Brodziak et al. 2021; Chun et al. 2021; de Sousa et al. 2022) data on cancer patients’ acceptance of vaccination against SARS-CoV-2. The rate of acceptance of SARS-CoV-2 vaccination in our cohort exceeds the German national average that has been previously reported at 60–70% (Neumann-Böhme et al. 2020; Lindholt et al. 2021).

In our cohort, fear of COVID-19 appears to be the main driver to receive vaccination, while on the other hand concerns about vaccine safety and possible side effects are regularly expressed. Nonetheless, SARS-CoV-2 vaccinations were well tolerated, side effects were scarce, and no serious adverse events occurred. In addition, almost no interference with the scheduling of anti-cancer therapy was documented.

After SARS-CoV-2 vaccination, most patients felt safe enough to resume parts of their personal activities, mostly meeting up with friends and family and everyday tasks such as shopping. This perception matches with study results indicating adequate protection for most patients after successful immunization (Addeo et al. 2021; Thakkar et al. 2021; Giuliano et al. 2022). In addition, the majority of patients indicated a willingness to receive repeated vaccinations against SARS-CoV-2, if necessary. This might indeed be necessary due to fading levels of (neutralizing) SARS-CoV-2 antibodies, especially among cancer patients and in respect to variants of concern (Fendler et al. 2022; Obeid et al. 2022). In fact, national institutions presently recommend second booster vaccination for vulnerable patient groups 3 months after the first booster vaccination (Bar-On et al. 2022; Koch et al. 2022).

Importantly, we found significant differences between male and female patients regarding both fear of COVID-19 or vaccine side effects as well as perceived safety after vaccination. Furthermore, tolerability of SARS-CoV-2 vaccination appears to be significantly associated with individual perception of the vaccine’s safety. This matches a recent review by Amanzio et al. (2022) about nocebo effects in COVID-19 vaccines and adds to previous studies reporting a higher tendency towards vaccine hesitancy among women (Neumann-Böhme et al. 2020; Latkin et al. 2021; Ogilvie et al. 2021). We, therefore, suggest evaluation of gender specific and sensitive information of patients.

While this is just a single-center experience, our study still features a reasonable number of participants from a wide range of oncological diseases. Additionally, this is to our knowledge the first study to report attitudes towards SARS-CoV-2 vaccination in our area depending on cancer site and gender as well as patients’ reports on subjectively perceived safety after vaccination.

Finally, concerns about vaccine safety remain an issue. Those as well as gender differences need to be addressed to increase vaccination rates and tolerability and to fight vaccine hesitancy. A recent study demonstrated that a large number of clinical trials on COVID-19 did not adequately include sex and gender in their study design (Brady et al. 2021). The present results may help identify patients that benefit from more detailed pre-vaccination consultation.

## Data Availability

The data used and analyzed during this study are available from the corresponding author upon reasonable request.

## References

[CR1] Addeo A, Shah PK, Bordry N, Hudson RD, Albracht B, Di Marco M, Kaklamani V, Dietrich PY, Taylor BS, Simand PF, Patel D, Wang J, Labidi-Galy I, Fertani S, Leach RJ, Sandoval J, Mesa R, Lathrop K, Mach N, Shah DP (2021). Immunogenicity of SARS-CoV-2 messenger RNA vaccines in patients with cancer. Cancer Cell.

[CR2] Amanzio M, Mitsikostas DD, Giovannelli F, Bartoli M, Cipriani GE, Brown WA (2022). Adverse events of active and placebo groups in SARS-CoV-2 vaccine randomized trials: a systematic review. Lancet Reg Health Eur.

[CR3] Baden LR, El Sahly HM, Essink B, Kotloff K, Frey S, Novak R, Diemert D, Spector SA, Rouphael N, Creech CB, McGettigan J, Khetan S, Segall N, Solis J, Brosz A, Fierro C, Schwartz H, Neuzil K, Corey L, Gilbert P, Janes H, Follmann D, Marovich M, Mascola J, Polakowski L, Ledgerwood J, Graham BS, Bennett H, Pajon R, Knightly C, Leav B, Deng W, Zhou H, Han S, Ivarsson M, Miller J, Zaks T, C. S. Group (2021). Efficacy and Safety of the mRNA-1273 SARS-CoV-2 Vaccine. N Engl J Med.

[CR4] Bar-On YM, Goldberg Y, Mandel M, Bodenheimer O, Amir O, Freedman L, Alroy-Preis S, Ash N, Huppert A, Milo R (2022). Protection by a fourth dose of BNT162b2 against Omicron in Israel. N Engl J Med.

[CR5] Barrière J, Gal J, Hoch B, Cassuto O, Leysalle A, Chamorey E, Borchiellini D (2021). Acceptance of SARS-CoV-2 vaccination among French patients with cancer: a cross-sectional survey. Ann Oncol.

[CR6] Bendau A, Plag J, Petzold MB, Ströhle A (2021). COVID-19 vaccine hesitancy and related fears and anxiety. Int Immunopharmacol.

[CR7] Brady E, Nielsen MW, Andersen JP, Oertelt-Prigione S (2021). Lack of consideration of sex and gender in COVID-19 clinical studies. Nat Commun.

[CR8] Brodziak A, Sigorski D, Osmola M, Wilk M, Gawlik-Urban A, Kiszka J, Machulska-Ciuraj K, Sobczuk P (2021). Attitudes of patients with cancer towards vaccinations-results of online survey with special focus on the vaccination against COVID-19. Vaccines (basel).

[CR9] Chun JY, Kim SI, Park EY, Park SY, Koh SJ, Cha Y, Yoo HJ, Joung JY, Yoon HM, Eom BW, Park CM, Han JY, Kim M, Lee DW, Kim JW, Keam B, Lee M, Kim TM, Choi YJ, Chang YJ, Lim MC (2021). Cancer patients' willingness to take COVID-19 vaccination: a nationwide multicenter survey in Korea. Cancers (basel).

[CR10] Dai M, Liu D, Liu M, Zhou F, Li G, Chen Z, Zhang Z, You H, Wu M, Zheng Q, Xiong Y, Xiong H, Wang C, Chen C, Xiong F, Zhang Y, Peng Y, Ge S, Zhen B, Yu T, Wang L, Wang H, Liu Y, Chen Y, Mei J, Gao X, Li Z, Gan L, He C, Shi Y, Qi Y, Yang J, Tenen DG, Chai L, Mucci LA, Santillana M, Cai H (2020). Patients with cancer appear more vulnerable to SARS-COV-2: a multi-center study during the COVID-19 outbreak. Cancer Discov.

[CR11] de Sousa MJ, Caramujo C, Júlio N, Magalhães JC, Basto R, Fraga T, Gomes IF, Monteiro AR, Pazos I, Sousa G (2022). Acceptance of SARS-CoV-2 vaccination among cancer patients in Portugal: attitudes and associated factors. Support Care Cancer.

[CR12] Fendler A, de Vries EGE, GeurtsvanKessel CH, Haanen JB, Wörmann B, Turajlic S, von Lilienfeld-Toal M (2022). COVID-19 vaccines in patients with cancer: immunogenicity, efficacy and safety. Nat Rev Clin Oncol.

[CR13] Giuliano AR, Lancet JE, Pilon-Thomas S, Dong N, Jain AG, Tan E, Ball S, Tworoger SS, Siegel EM, Whiting J, Mo Q, Cubitt CL, Dukes CW, Hensel JA, Keenan RJ, Hwu P (2022). Evaluation of antibody response to SARS-CoV-2 mRNA-1273 vaccination in patients with cancer in Florida. JAMA Oncol.

[CR14] Heyerdahl LW, Vray M, Lana B, Tvardik N, Gobat N, Wanat M, Tonkin-Crine S, Anthierens S, Goossens H, Giles-Vernick T (2022). Conditionality of COVID-19 vaccine acceptance in European countries. Vaccine.

[CR15] Heyne S, Esser P, Werner A, Lehmann-Laue A, Mehnert-Theuerkauf A (2022). Attitudes toward a COVID-19 vaccine and vaccination status in cancer patients: a cross-sectional survey. J Cancer Res Clin Oncol.

[CR16] Holzmann-Littig C, Braunisch MC, Kranke P, Popp M, Seeber C, Fichtner F, Littig B, Carbajo-Lozoya J, Allwang C, Frank T, Meerpohl JJ, Haller B, Schmaderer C (2021). COVID-19 vaccination acceptance and hesitancy among healthcare workers in Germany. Vaccines (basel).

[CR17] Ioannidis JPA (2020). Global perspective of COVID-19 epidemiology for a full-cycle pandemic. Eur J Clin Invest.

[CR18] Jaiswal J, LoSchiavo C, Perlman DC (2020). Disinformation, misinformation and inequality-driven mistrust in the time of Covid-19: lessons unlearned from AIDS denialism. AIDS Behav.

[CR19] Koch J, Vygen-Bonnet S, Bogdan C, Burchard G, Garbe E, Heininger U, Hummers E, Kling K, von Kries R, Ledig T, Littmann M, Meerpohl J, Mertens T, Meyer H, Perumal N, Röbl-Mathieu M, van der Sande M, Schönfeld V, Steffen A, Terhardt M, Überla K, Wichmann O, Wicker S, Wiedermann-Schmidt U, Widders G, Zepp F (2022). STIKO-Empfehlung zur 2. COVID-19-Auffrischimpfung mit einem mRNA- Impfstoff für besonders gesundheitlich gefährdete bzw. exponierte Personengruppen und die dazugehörige wissenschaftliche Begründung. Epid Bull.

[CR20] Latkin CA, Dayton L, Yi G, Colon B, Kong X (2021). Mask usage, social distancing, racial, and gender correlates of COVID-19 vaccine intentions among adults in the US. PLoS ONE.

[CR21] Lee LYW, Cazier JB, Starkey T, Briggs SEW, Arnold R, Bisht V, Booth S, Campton NA, Cheng VWT, Collins G, Curley HM, Earwaker P, Fittall MW, Gennatas S, Goel A, Hartley S, Hughes DJ, Kerr D, Lee AJX, Lee RJ, Lee SM, Mckenzie H, Middleton CP, Murugaesu N, Newsom-Davis T, Olsson-Brown AC, Palles C, Powles T, Protheroe EA, Purshouse K, Sharma-Oates A, Sivakumar S, Smith AJ, Topping O, Turnbull CD, Várnai C, Briggs ADM, Middleton G, Kerr R, U. C. C. M. P. Team (2020). COVID-19 prevalence and mortality in patients with cancer and the effect of primary tumour subtype and patient demographics: a prospective cohort study. Lancet Oncol.

[CR22] Lindholt MF, Jørgensen F, Bor A, Petersen MB (2021). Public acceptance of COVID-19 vaccines: cross-national evidence on levels and individual-level predictors using observational data. BMJ Open.

[CR23] Neumann-Böhme S, Varghese NE, Sabat I, Barros PP, Brouwer W, van Exel J, Schreyögg J, Stargardt T (2020). Once we have it, will we use it? a European survey on willingness to be vaccinated against COVID-19. Eur J Health Econ.

[CR24] Obeid M, Suffiotti M, Pellaton C, Bouchaab H, Cairoli A, Salvadé V, Stevenel C, Hottinger R, Pythoud C, Coutechier L, Molinari L, Trono D, Ribi C, Gottardo R, Fenwick C, Pascual M, Duchosal MA, Peters S, Pantaleo G (2022). Humoral responses against variants of concern by COVID-19 mRNA vaccines in immunocompromised patients. JAMA Oncol.

[CR25] Ogilvie GS, Gordon S, Smith LW, Albert A, Racey CS, Booth A, Gottschlich A, Goldfarb D, Murray MCM, Galea LAM, Kaida A, Brotto LA, Sadarangani M (2021). Intention to receive a COVID-19 vaccine: results from a population-based survey in Canada. BMC Public Health.

[CR26] Overheu O, Quast DR, Schmidt WE, Sakinç-Güler T, Reinacher-Schick A (2022). Low serological prevalence of SARS-CoV-2 antibodies in cancer patients at a German university oncology center. Oncol Res Treat.

[CR27] Pertwee E, Simas C, Larson HJ (2022). An epidemic of uncertainty: rumors, conspiracy theories and vaccine hesitancy. Nat Med.

[CR28] Polack FP, Thomas SJ, Kitchin N, Absalon J, Gurtman A, Lockhart S, Perez JL, Pérez Marc G, Moreira ED, Zerbini C, Bailey R, Swanson KA, Roychoudhury S, Koury K, Li P, Kalina WV, Cooper D, Frenck RW, Hammitt LL, Türeci Ö, Nell H, Schaefer A, Ünal S, Tresnan DB, Mather S, Dormitzer PR, Şahin U, Jansen KU, Gruber WC, C. C. T. Group (2020). Safety and Efficacy of the BNT162b2 mRNA Covid-19 Vaccine. N Engl J Med.

[CR29] Rüthrich MM, Giessen-Jung C, Borgmann S, Classen AY, Dolff S, Grüner B, Hanses F, Isberner N, Köhler P, Lanznaster J, Merle U, Nadalin S, Piepel C, Schneider J, Schons M, Strauss R, Tometten L, Vehreschild JJ, von Lilienfeld-Toal M, Beutel G, Wille K, L. S. Group (2021). COVID-19 in cancer patients: clinical characteristics and outcome-an analysis of the LEOSS registry. Ann Hematol.

[CR30] Shakeel CS, Mujeeb AA, Mirza MS, Chaudhry B, Khan SJ (2022). Global COVID-19 Vaccine acceptance: a systematic review of associated social and behavioral factors. Vaccines (basel).

[CR31] Thakkar A, Gonzalez-Lugo JD, Goradia N, Gali R, Shapiro LC, Pradhan K, Rahman S, Kim SY, Ko B, Sica RA, Kornblum N, Bachier-Rodriguez L, McCort M, Goel S, Perez-Soler R, Packer S, Sparano J, Gartrell B, Makower D, Goldstein YD, Wolgast L, Verma A, Halmos B (2021). Seroconversion rates following COVID-19 vaccination among patients with cancer. Cancer Cell.

[CR32] Thunström L, Ashworth M, Finnoff D, Newbold SC (2021). Hesitancy toward a COVID-19 vaccine. EcoHealth.

[CR33] Umakanthan S, Lawrence S (2022). Predictors of COVID-19 vaccine hesitancy in Germany: a cross-sectional, population-based study. Postgrad Med J.

[CR34] Voysey M, Clemens SAC, Madhi SA, Weckx LY, Folegatti PM, Aley PK, Angus B, Baillie VL, Barnabas SL, Bhorat QE, Bibi S, Briner C, Cicconi P, Collins AM, Colin-Jones R, Cutland CL, Darton TC, Dheda K, Duncan CJA, Emary KRW, Ewer KJ, Fairlie L, Faust SN, Feng S, Ferreira DM, Finn A, Goodman AL, Green CM, Green CA, Heath PT, Hill C, Hill H, Hirsch I, Hodgson SHC, Izu A, Jackson S, Jenkin D, Joe CCD, Kerridge S, Koen A, Kwatra G, Lazarus R, Lawrie AM, Lelliott A, Libri V, Lillie PJ, Mallory R, Mendes AVA, Milan EP, Minassian AM, McGregor A, Morrison H, Mujadidi YF, Nana A, O'Reilly PJ, Padayachee SD, Pittella A, Plested E, Pollock KM, Ramasamy MN, Rhead S, Schwarzbold AV, Singh N, Smith A, Song R, Snape MD, Sprinz E, Sutherland RK, Tarrant R, Thomson EC, Török ME, Toshner M, Turner DPJ, Vekemans J, Villafana TL, Watson MEE, Williams CJ, Douglas AD, Hill AVS, Lambe T, Gilbert SC, Pollard AJ, O. C. V. T. Group (2021). Safety and efficacy of the ChAdOx1 nCoV-19 vaccine (AZD1222) against SARS-CoV-2: an interim analysis of four randomised controlled trials in Brazil, South Africa, and the UK. Lancet.

[CR35] Williamson EJ, Walker AJ, Bhaskaran K, Bacon S, Bates C, Morton CE, Curtis HJ, Mehrkar A, Evans D, Inglesby P, Cockburn J, McDonald HI, MacKenna B, Tomlinson L, Douglas IJ, Rentsch CT, Mathur R, Wong AYS, Grieve R, Harrison D, Forbes H, Schultze A, Croker R, Parry J, Hester F, Harper S, Perera R, Evans SJW, Smeeth L, Goldacre B (2020). Factors associated with COVID-19-related death using OpenSAFELY. Nature.

[CR36] World Health Organization (WHO, 2019) Ten threats to global health in 2019., from https://www.who.int/news-room/spotlight/ten-threats-to-global-health-in-2019.

[CR37] Wu F, Zhao S, Yu B, Chen YM, Wang W, Song ZG, Hu Y, Tao ZW, Tian JH, Pei YY, Yuan ML, Zhang YL, Dai FH, Liu Y, Wang QM, Zheng JJ, Xu L, Holmes EC, Zhang YZ (2020). A new coronavirus associated with human respiratory disease in China. Nature.

